# Age-related changes in the quadriceps tendon: Collagen fibril diameter decreases with aging

**DOI:** 10.1016/j.asmart.2025.06.002

**Published:** 2025-07-04

**Authors:** Yoshihiro Ishida, Yasushi Takata, Tatsuya Ishikawa, Mitsuhiro Kimura, Naoki Takemoto, Manase Nishimura, Noriyuki Ozaki, Satoru Demura, Junsuke Nakase

**Affiliations:** a Department of Orthopedic Surgery, Graduate School of Medical Science, Kanazawa University, Kanazawa, Japan; b Department of Functional Anatomy, Graduate School of Medical Science, Kanazawa University, 13-1 Takara-machi, 920-8640, Kanazawa, Japan; c Department of Orthopedic Surgery, Fukui General Hospital, 58-16-1, Egami-Town, Fukui, 910-8561, Japan

**Keywords:** Aging, Collagen fibril diameter, Graft, Quadriceps tendon

## Abstract

**Background:**

The quadriceps tendon (QT) has recently gained attention as a graft tendon for anterior cruciate ligament (ACL) reconstruction due to its high strength from a larger ultrastructural collagen fibril diameter in children than that of the semitendinosus tendon. While QT collagen fibril diameter increases with growth, changes in older adults remain unclear. This study investigated age-related changes in QT collagen fibril diameter.

**Methods:**

Twenty-four patients who had undergone ACL reconstruction using the QT or other knee surgeries were included. QT tissues collected during surgery were analyzed using transmission electron microscopy. Collagen fibril diameter was measured in four groups: Immature (11–13 years), Young (15–17 years), Adult (21–50 years), and Older (61–81 years). The average fibril diameter was calculated for each sample. At least four slides (one slide = one digital electron micrographs of the ultrathin section) were evaluated for each specimen, with at least 200 collagen fibrils on each slide. The average number of collagen fibrils measured per specimen was 812 ± 10. Data of the four groups were analyzed using one-way analysis of variance and Tukey's test.

**Results:**

The mean collagen fibril diameters were 89.7 ± 14.4, 94.8 ± 16.4, 107.2 ± 12.1, and 73.0 ± 9.7 nm in the Immature, Young, Adult, and Older groups, respectively. Although no significant difference was observed between the Immature and Young groups, fibril diameter was significantly larger in the Adult group than in the Young group and significantly smaller in the Older group than in the other groups.

**Conclusions:**

The average QT collagen fibril diameter increased with growth but was lower in the Older group, suggesting a decrease with aging.

## 1 Introduction

The incidence of anterior cruciate ligament (ACL) rupture has increased in recent years^1^ due to several factors, including an increase in the number of people participating in sports and efforts to improve health across generations. For young and active patients, ACL reconstruction is recommended to prevent secondary damage to the meniscus and cartilage.^2^^,^^3^ However, problems such as a high rate of postoperative complications in patients aged >50 years, among other issues, need to be considered.^4^

The hamstring tendon (HT), bone–patellar tendon–bone, and recently, the quadriceps tendon (QT) have been commonly used as graft tendons for ACL reconstruction. However, the QT has attracted attention due to its excellent biomechanical properties and low incidence of harvest site complications.^5^, ^6^, ^7^, ^8^, ^9^ On the other hand, HT has been shown to have disadvantages, such as a higher revision rate in children than in adults.^10^ QT has superior rigidity, and has a lower revision rate compared to HT.^11^,^12^ It is expected that QT will be not only in pediatric patients but also in athletes who play contact sports.

To examine the factors contributing to higher revision rate in ACL reconstruction using the HT in children whose bones have not yet matured, Asai et al. investigated the histological and ultrastructural properties of the HT as important factors influencing the mechanical properties of tendons and found that the diameter of the collagen fibrils in the HT gradually increased with growth.^13^ Furthermore, a previous study by Kimura et al. showed that the diameter of QT collagen fibrils increased with growth.^14^ However, how the diameter of collagen fibrils change in adults aged ≥60 years remains unclear. Therefore, this study aimed to investigate the influence of aging on the ultrastructural properties of the QT. Previous studies have reported that tenofibril density decreases with age^15^ and aged tendon fibroblasts and actin cytoskeleton become poorly organized.^16^ Furthermore, in experiments in rabbit models, the area and diameter of collagen fibrils in the soleus and lateral gastrocnemius tendons were significantly larger at 8–10 months of age than at 3 weeks of age and were smaller at 4–5 years of age.^17^ We hypothesized that collagen tendon fibril diameter of the QT would be reduced in older adults compared with that in younger adults.

## 2 Material and methods

### 2.1 Sample preparation

This study was performed in accordance with the ethical standards of the 1964 Declaration of Helsinki and its later amendments or comparable ethical standards. The study design was approved by the Ethics Committee of Kanazawa university hospital (approval no. 113893). All patients were informed about the purpose, procedures, and known risks of the techniques, and they provided written informed consent.

Twenty-four patients who had undergone ACL reconstruction using QT or other surgeries around the knee joint between March 2021 and July 2023 were included. Tissue samples (5 × 3 mm) were obtained from the central portion of the tendon, 1 cm proximal to the patellar attachment. For conformance with ethical standards, only the surplus tissue discarded during trimming of tendons for autografting was used, and no new incisions were made for sample collection.

On T2-weighted fat-suppressed coronal magnetic resonance imaging (MRI), an open femoral epiphyseal plate was defined as an epiphyseal plate thickness >1.5 mm in the median femur. Subsequently, patients were classified into four groups according to age and epiphyseal plate status: the “Immature” group comprised patients (mean age±standard deviation [SD], 11.5 ± 1.6 years) with open epiphyseal plates confirmed using MRI, “Young” group comprised patients aged <20 years (15.8 ± 1.0 years) with closed epiphyseal plates, “Adult” group comprised patients aged >20 years (29.8 ± 11.3 years old) irrespective of their epiphyseal plate status,^16^ and “Older” group comprised patients aged >60 years (70.3 ± 9.7 years old). Patients were included irrespective of the presence of osteoarthritis, but Patients with a history of trauma or surgery around the knee joint were excluded.

### 2.2 Estimation of the size and distribution of collagen fibrils

The diameters of collagen fibrils in the QT for the Immature, Young, and Adult groups were obtained from the data of 18 patients included in a previous study by Kimura et al..^14^ Ultrastructural analysis of the samples was performed using transmission electron microscopy (H-7650; Hitachi High-Technologies Co., Tokyo, Japan). Tendon tissues were sampled and sectioned into small pieces using a blade, and they were fixed in 2 % paraformaldehyde (Sigma-Aldrich; St Louis, MO, USA) and 2.5 % glutaraldehyde (FUJIFILM Wako Pure Chemical, Osaka, Japan) in 0.1 M phosphate buffer (PB, pH 7.4) overnight at 4 °C. These sections were incubated in a fixative containing 2 % osmium tetroxide for 60 min on ice and counterstained en bloc with 1 % uranyl acetate overnight at 4 °C. The tendons were dehydrated by sequential treatment of 50, 70, 80, 90, 95, and 100 % ethanol for 10 min each, placed in the second solution of 100 % ethanol, and incubated twice in n-Butyl glycidyl ether (QY-1; NISSHIN EM, Tokyo, Japan). These samples were then incubated in a 1:1 mixture of QY-1/Quetol-812 resin (NISSHIN EM) for 10 min and embedded overnight at room temperature in Quetol-812. After resin curing at 60 °C for 2 days, ultrathin sections of 70-nm thickness were obtained using an ultramicrotome (Ultracut-T; Leica, Wetzlar, Germany). These sections were collected onto copper grids (NISSHIN EM) and contrasted with lead citrate for 5 min. The samples were examined at 8000 × or 12000 × magnification using a transmission electron microscope (H-7650; Hitachi High-Technologies Co., Tokyo, Japan). Digital electron micrographs of the ultrathin sections were obtained.

**Fig. 1:**
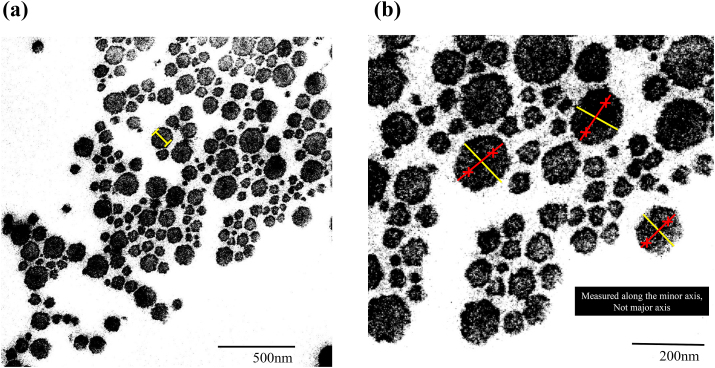
As the collagen fibrils in tendons are cylindrical state, these exhibit an elliptical morphology when cut. Therefore, the minimum diameter(minor axis) of each fibril can be used to estimate the true fibril diameter.

From these cross-sections, the minimal collagen fibril diameter was measured to the nearest 0.1 mm using Image J (National Institutes of Health, Bethesda, MD, USA). As the fibrils are cylindrical, collagen fibrils in tendons exhibit an elliptical morphology when cut obliquely. Therefore, the minimum diameter of each fibril can be used to estimate the true fibril diameter (Fig. 1). Fibrils showing a banding pattern were excluded. At least four slides (one slide = one digital electron micrographs of the ultrathin section) from each sample were evaluated, with at least 200 collagen fibrils on each slide. Finally, the average number of collagen fibrils per sample was 812 ± 10. The mean collagen fibril diameter of each sample and distribution of fibril diameters were compared among the four groups.

One investigator (Y.I.) obtained the samples and was the only one aware of group assignments. A second investigator (T.I.) prepared sections of each sample and observed them using electron microscopy. Comparative examination of patient data from the Immature, Young, Adult, and Older groups was then performed.

### 2.3 Statistical analysis

All measurements are presented as mean ± SD and were analyzed using SPSS software (version 24.0; IBM Corp., Armonk, NY, USA). After testing for normal distribution using the Shapiro–Wilk test, one-way analysis of variance and Tukey's honest significant difference test were used to compare the mean fibril diameter from each sample among the four groups. Based on the average number of collagen fibrils (n = 812), a histogram was created with 11 bins using Sturges' formula (k = 1 + log2N). The fibril diameter distribution was non-Gaussian, and the Kolmogorov–Smirnov test was used to compare the distribution of collagen fibrils among the four groups. In addition, the Bonferroni correction was used to account for false positives in multiple comparisons. The significance level set at p < 0.05 or p < 0.016 (0.05/3) after Bonferroni correction. The intra-observer reliability, obtained using the intraclass correlation coefficient for the measured collagen fibrils, was 0.956. Inter-observer reliability was 0.888. The recommended sample sizes were evaluated by computing the statistical power using G-Power 3.1 software (Heinrich-Heine University Düsseldorf, Düsseldorf, Germany), based on a previous study; a minimum of six samples per group (effect size 0.80, alpha error 0.05, target power 0.8) were recommended, as in previous studies.

## 3 Results

**Fig. 2:**
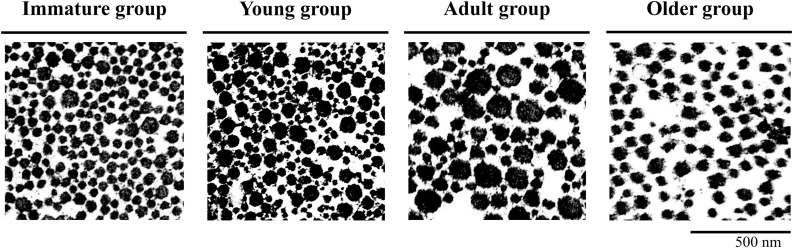
QT collagen fibril diameter increased with growth in the Immature, Young, and Adult groups but was lower in the Older group significantly. ※However, no significant differences were observed between Immature and Young group(p=0.607).

**Fig. 3:**
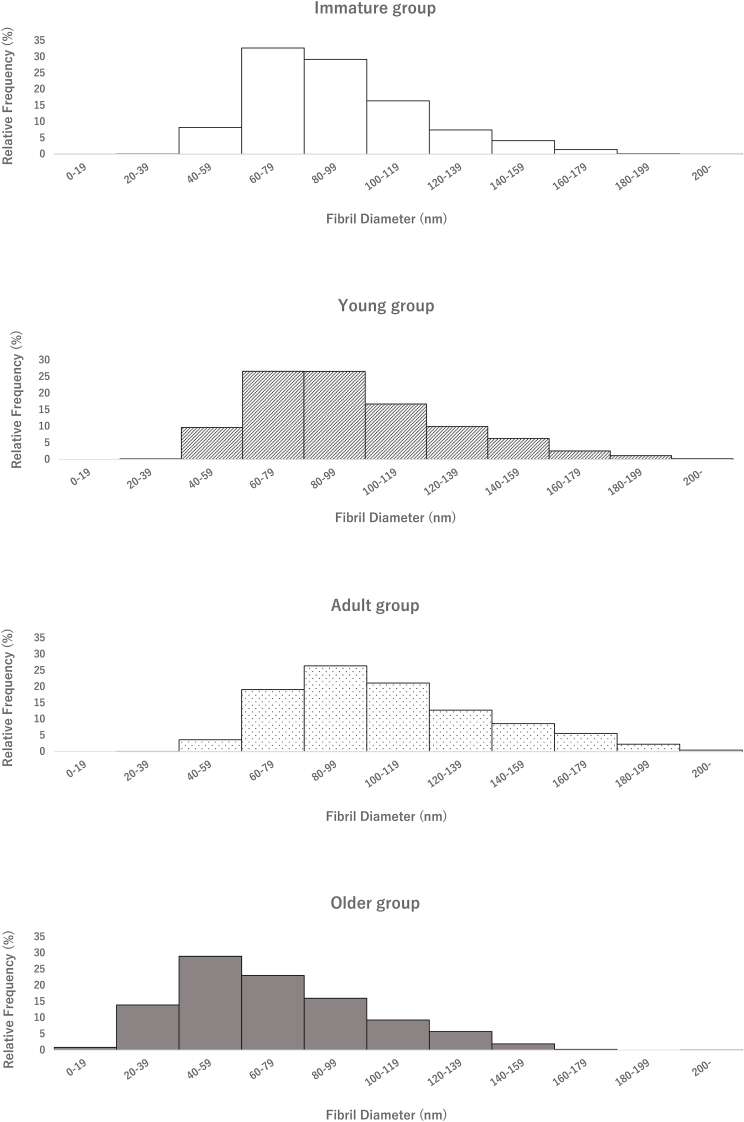
The histogram of the collagen fibril diameter changed from a right-skewed distribution to flat with growth from the immature group to the adult group, and in the elderly group, the overall distribution shifted to the left while maintaining a relatively flat trend.

Table 1 shows the characteristics patients in all groups. The average collagen fibril diameters were 89.7 ± 14.4 nm, 94.8 ± 16.4 nm, and 107.2 ± 12.1 nm in the Immature, Young, and Adult groups, respectively, and 73 ± 9.7 nm in the Older group (Table 1). Although no significant difference was observed between the Immature and Young groups, the average fibril diameter was significantly larger in the Adult group than in the Immature and Young groups and significantly smaller in the Older group than in the other three groups (Table 2). Fig. 2 shows photographs of representative collagen fibril diameters for each group. In the analysis of the non-Gaussian distribution of the minimum fibril diameter after Bonferroni correction, significant differences were observed between the Immature and Adult, Immature and Older, Young and Adult, Young and Older, and Adult and Older groups (p < 0.001), and Young and Adult group (p = 0.019). However, no significant differences were observed between Immature and Young group(p = 0.607). In addition, histograms were used to compare the peak positions and distribution shapes of fibril diameters by age groups, in order to examine whether only collagen fibrils with a specific diameter range were changing with age. The histogram of the collagen fibril diameter changed from a right-skewed distribution to flat with growth from the immature group to the adult group, and in the elderly group, the overall distribution shifted to the left while maintaining a relatively flat trend (Fig. 3).

**Table 1:** Data are presented as mean ± standard deviation.

	Immature group	Young group	Adult group	Older group
Case (n)	6	6	6	6
Age (years)	11-13 (12.3 ± 0.8)	15-17 (15.8 ± 1.0)	21-50 (29.8 ± 11.3)	61-81 (73.0 ± 7.0)
Sex (male:female)	4:2	4:2	3:3	0:6
Fibril diameter (nm)	89.7 ± 14.4	94.4 ± 16.4	107.2 ± 12.1	70.3 ± 9.3

Case Details						
Group	Case	Age (years)	Sex	Height (cm)	Sports	Fibril diameter (nm)
Immature	1	13	male	149.5	basket	80.3
	2	11	male	131.2	baseball	103.5
	3	13	male	125.2	hokey	88.8
	4	13	female	128.3	soccer	107.53
	5	12	male	144.5	baseball	68.78
	6	13	female	148.3	basketball	89.18
Young	1	15	male	179.5	basketball	102.85
	2	17	male	165.0	baseball	65.58
	3	16	male	169.3	baseball	93.73
	4	15	male	160.5	baseball	98.26
	5	15	male	179.5	baseball	115.19
	6	17	female	162.3	ski	93.37
Adult	1	22	female	157.5	softball	104.75
	2	50	female	167.9	tennis	86.56
	3	31	female	158.0	yoga	104.01
	4	21	male	175.0	soccer	116.84
	5	34	male	178.3	basketball	121.1
	6	21	male	170.0	baseball	110.17
Older	1	68	female	160.6	none	62.51
	2	81	female	142.0	none	57.16
	3	78	female	139.1	none	76.69
	4	71	female	153.0	none	78.78
	5	69	female	152.0	none	63.07
	6	61	female	144.4	none	62.51

**Table 2:** One-way analysis of variance and Tukey’s honest significant difference test were used. The significance level was set at p<0.05.

Group	p	MD	SE	95 % CI
Immature vs. Young	0.607	−5.15	4.17	[-16.1, 5.8]
Immature vs. Adult	<0.001	−17.6	4.17	[-28.5, −6.6]
Immature vs. Older	<0.001	19.4	4.17	[8.5, 30.3]
Young vs. Adult	0.019	−12.4	4.17	[-23.3, −1.5]
Young vs. Older	<0.001	24.6	4.17	[13.6, 35.5]
Adult vs. Older	<0.001	37.0	4.17	[26.1, 47.9]

## 4 Discussion

In this study, we investigated the mean collagen fibril diameter and the distribution in four age groups. There is so much difference in fibril diameter among the Young group in the study. I think that this is because this is the age group in which collagen fibrils are in the growth stage.

An important finding of this study is that QT collagen fibril diameter increased with growth in the Immature, Young, and Adult groups but was lower in the Older group, suggesting a decrease with aging. In addition, the histogram of collagen fibril diameters changed from a right-skewed distribution to flat with growth from the immature group to the adult group, and in the elderly group, the overall distribution shifted to the left while maintaining a relatively flat trend. The elderly group had a flat distribution of fiber diameters, and contained diameters smaller than those in the immature group. These results indicate that there is an age-dependent bias in the distribution of collagen fibril diameters, that the average fibril diameter increases from the immature group to adulthood while the distribution becomes flat, and that from adulthood onwards, all sizes of collagen fibrils shrink with age.

A previous report showed that the collagen fibril diameter of the QT changes with growth, similarly to the semitendinosus tendon (ST). However, in all cases, the fibril diameter increased with age and did not decrease further.^13^^,^^14^ Furthermore, whether collagen fibril diameter changes with age in human tendon tissues that have completed their growth phase remains unclear. Therefore, the results of this study may provide more detailed knowledge on human developmental processes.

Although the biological mechanisms that promote tendon maturation are mostly unknown, human studies have shown that a loss of mechanical stimulation of the tendon reduces cross-linking within collagen structures.^19^^,^^20^ Further, fewer blood vessels and decreased blood flow have been reported in older patients compared with that in younger patients.^21^ In addition, aging tendons undergo fat and cartilage formation and hardening, impairing tendon structure and function.^18^^,^^22^, ^23^, ^24^ Animal experiments have also shown that the density of tendon cells decreases with age in rabbits and rats.^25^, ^26^, ^27^ The area and diameter of collagen fibrils in the rabbit soleus and lateral gastrocnemius tendons also decrease with age; these were significantly higher at 8–10 months of age than at 3 weeks but lower in older animals.^17^ In addition, it is known that advanced glycation end products (AGEs), which are associated with aging and lifestyle-related diseases in humans, accumulate in long-lived proteins such as collagen with age, resulting in significantly higher concentrations in older people than in younger people.^28^, ^29^, ^30^

Based on these findings, in older humans, tissue-wide changes may occur in the collagen structure of tendons due to decreased physical activity and age-related changes inside and outside cells.

Tendon strength and the collagen fibril radius are positively correlated.^31^ Decreases in collagen fibril diameter with aging may, thus, reduce the strength of grafted tendons and increase the risk of re-rupture after ACL reconstruction.

Regarding this study's clinical significance, a review comparing the clinical outcomes of ACL reconstruction between patients aged at least 50 years and those younger than 50 years concluded that there were no significant differences in Lysholm and Tegner scores or anterior–posterior knee instability. However, it has been reported that 1.6 % of patients aged at least 50 years underwent total knee arthroplasty within 2 years.^4^ Currently, there is no clear consensus regarding the appropriate age for ACL reconstruction surgery or the selection of graft tendons depending on patient age. For optimal treatment selection, orthopedic surgeons must consider graft stability and durability, as well as age-related tissue changes.

In ACL reconstruction, the re-rupture rate with allograft tendons is significantly higher than that with autografts.^22^ In a biomechanical study that investigated the strength of transplanted tendons in donors aged between 49 and 99 years, aging had a negative effect on the elastic modulus and maximum tensile force.^23^ These findings may explain the poor outcomes observed with tendon allografts.

This study has some limitations. First, the sample size was small and limited by the number of cases. In addition, all six patients in the Older group were women, indicating a gender bias. Second, the evaluation of graft samples was restricted to a single site; thus, whether these are representative of the entire graft remains unclear. Particularly, samples were taken from the center of the graft; samples from the ends of the graft or other sites were not evaluated. Third, five of the six patients in the Older group had severe knee osteoarthritis and required knee replacement surgery, suggesting that their knee condition was different from that of a healthy older adult.

In the future, we hope to analyze a larger number of cases to establish more reliable data on fibril diameter that is not influenced by gender bias or underlying diseases.

## 5 Limitation

A limitation of this study is the large age difference between the adult and Older groups. Although the prognosis of ACL reconstruction is sometimes discussed in relation to patients over the age of 50, the results of this study may not be applicable to patients in their 50s, as there is insufficient data in this age group.

## 6 Conclusions

This study showed that the diameter of QT collagen fibrils, which increases with skeletal growth, was significantly reduced in the Older group. These findings provide new foundational medical knowledge about age-related changes in human tendons. They are expected to be useful in the treatment of older patients undergoing ACL reconstruction surgery by providing insights about grafts and aiding in prognosis, thus informing clinicians on the possibility of re-rupture.

## Author contributions

Writing - review & editing: Yoshihiro Ishida, Yasushi Takata, Tatsuya Ishikawa, Mitsuhiro Kimura, Naoki Takemoto, Manase Nishimura, Noriyuki Ozaki, Satoru Demura, Junsuke Nakase.

Experimenting & analyzing: Yoshihiro Ishida, Tatsuya Ishikawa, Mitsuhiro Kimura.

## Funding

None.

## Declaration of competing interest

No conflicts of interests are declared.

We attest that authors have not received or will not receive benefits for personal or professional use from a commercial party related directly or indirectly to the subject of this article.
